# Stent-Assisted Coiling of Unruptured MCA Aneurysms Using the LVIS Jr. Device: A Multicenter Registry

**DOI:** 10.3390/jcm9103168

**Published:** 2020-09-30

**Authors:** Wojciech Poncyljusz, Łukasz Zwarzany, Bartosz Limanówka, Miłosz Zbroszczyk, Mariusz Banach, Sławomir Bereza, Leszek Sagan

**Affiliations:** 1 Department of Diagnostic Imaging and Interventional Radiology, Pomeranian Medical University, Unii Lubelskiej 1, 71-252 Szczecin, Poland; wponcyl@poczta.onet.pl; 2 Department of Neurosurgery and Pediatric Neurosurgery, Pomeranian Medical University, Unii Lubelskiej 1, 71-252 Szczecin, Poland; blimanowka@gmail.com (B.L.); leszekm.sagan@gmail.com (L.S.); 3 Department of Radiodiagnostic and Interventional Radiology, Silesian Medical University, Medyków 14, 40-772 Katowice, Poland; miloszz@wp.pl; 4 Department of Neurosurgery, Saint Raphael Hospital, Adama Bochenka 12, 30-693 Kraków, Poland; marban13@gmail.com; 5 Neurointerventional CathLab, Lower Silesian Specialist Tadeusz Marciniak Memorial Hospital—Emergency Medicine Center, Fieldorfa 2, 54-049 Wrocław, Poland; slawomir.bereza@gmail.com

**Keywords:** intracranial aneurysm, middle cerebral artery, stent-assisted coiling, low-profile stent, braided stent

## Abstract

Purpose: The low-profile visualized intraluminal support junior (LVIS Jr.) is a new generation low-profile braided stent. Our aim was to evaluate the safety and efficacy of the LVIS Jr. in the stent-assisted coiling of unruptured middle cerebral artery (MCA) aneurysms. This was a multicenter retrospective study. Patient demographics, aneurysm characteristics, procedural details, complications, and the results of clinical and imaging follow-up were analyzed. Four centers participated in the study. A total of 162 consecutive patients with 162 unruptured MCA aneurysms were included for the analysis. The mean aneurysm size was 7.6 mm (range 2 to 37 mm) and 97.5% were wide-necked. Immediate postprocedural angiograms showed Raymond-Roy class 1 in 118 (72.8%), class 2 in 23 (14.2%), and class 3 in 21 patients (13%). Periprocedural complications occurred in 14 patients (8.6%). There were no procedure-related deaths. Follow-up imaging at 12–18 months post-procedure showed Raymond–Roy class 1 in 132 (81.5%), class 2 in 17 (10.5%), and class 3 in 13 patients (8%). There were 3 cases of in-stent stenosis (1.9%). All 162 patients had good clinical outcome (mRS score 0–2) at 90 days post-procedure. Stent-assisted coiling of unruptured MCA aneurysms with the LVIS Jr. stent is safe and effective, with high immediate and long-term total occlusion rates.

## 1. Introduction

Endovascular coiling is an established method for treating unruptured intracranial aneurysms [1,2,3,4,5]. However, when the aneurysm morphology is complex, it is often a technically challenging procedure, even for an experienced interventional radiologist. This is especially true for middle cerebral artery (MCA) aneurysms. These aneurysms, in many cases, are wide-necked and, commonly, the branch vessels arise from the aneurysm sac. The endovascular coiling of MCA aneurysms is associated with a higher complication rate when compared to the treatment of more proximal lesions [6].

**Figure 1:**
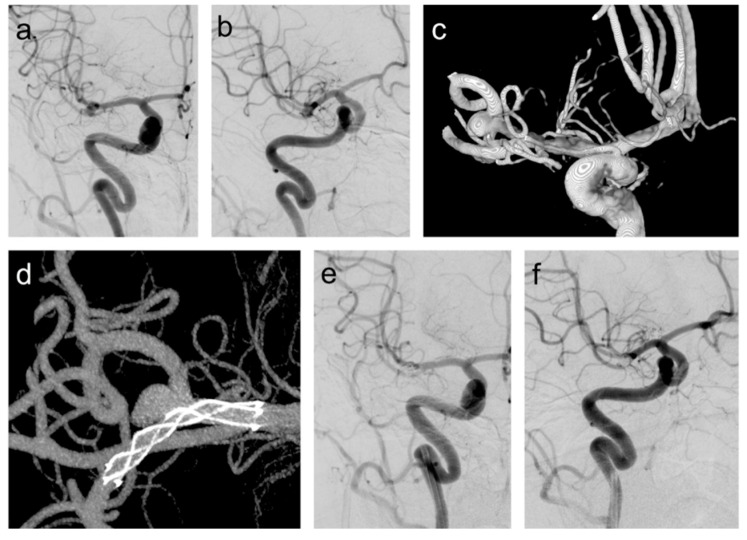
(**a**,**b**) A patient with a wide-necked right middle cerebral artery (MCA) bifurcation aneurysm. The aneurysm was treated with the stent-assisted coiling using the low-profile visualized intraluminal support junior (LVIS Jr.); (**c**) three-dimensional reconstruction demonstrates the complex morphology of the aneurysm; (**d**) intraprocedural contrast-enhanced flat panel detector computed tomography (CT) (maximum intensity projection (MIP) reconstruction) showing full stent opening with wall adherence along the length of the device; (**e**,**f**) Post-procedural digital subtraction angiography (DSA) showing complete occlusion of the aneurysm.

Due to the ongoing development of newer neurovascular devices more patients with intracranial aneurysms are eligible for minimally invasive endovascular treatment. A number of devices can be used to support the embolization of wide-necked aneurysms, including balloons, stents, and, more recently, the Woven EndoBridge (WEB) device (MicroVention-Terumo, Tustin, CA, USA) [7]. The low-profile visualized intraluminal support junior (LVIS Jr.; MicroVention-Terumo, Tustin, CA, USA) is a self-expanding, braided, and closed-cell nitinol microstent with compliant 1.5 mm cell size. The LVIS Jr. has three radiopaque proximal and distal markers, as well as three helical radiopaque strands within the body of the stent for complete visualization. The device is compatible with a 0.017-inch microcatheter and may be deployed in vessels sized 2.0–3.5 mm (Figure 1). We present the results from multicenter retrospective registry evaluating the safety and efficacy of the stent-assisted coiling technique for MCA unruptured aneurysm. All patients in the present study were treated exclusively with the LVIS Jr. stent.

## 2. Materials and Methods

Four tertiary care centers participated in this retrospective study, which was approved by the bioethics commission at the lead investigator’s site. Consecutive cases of unruptured MCA aneurysms treated with the stent-assisted coiling using the LVIS Jr. stent from January 2013 to December 2019 were included for the analysis. Patient demographics, aneurysm characteristics, procedural details, and complications were collected from the medical records.

Written informed consent for the procedure was obtained from all subjects. Dual antiplatelet therapy was administered for at least 5 days prior to the treatment (acetylsalicylic acid (Acard; Polfa Warszawa, Warsaw, Poland) in a dose of 150 mg/daily and clopidogrel (Plavix; Sanofi Aventis, Paris, France) in a dose of 75 mg/daily). All procedures were performed under general anesthesia and systemic heparinization by experienced interventional radiologists via the femoral approach using 6F Brite Tip sheath introducer (Cordis, Bridgewater, NJ, USA) and guiding catheter Chaperon 6F (MicroVention-Terumo, Tustin, CA, USA) or Guider Soft (Stryker, Fremont, CA, USA) which was positioned in the internal carotid artery (ICA). The Traxcess 0.014-inch microguidewire (MicroVention-Terumo, Tustin, CA, USA) or Terumo 0.012-inch, double angled, (Terumo Medical Corporation, Tokyo, Japan) and Headway 17 microcatheter (MicroVention-Terumo, Tustin, CA, USA) was used for deployment of the LVIS Jr. stent. Stent selection and deployment was performed in accordance with the manufacturer’s instructions. The jailing technique or coiling through the cells of the stents was dependent on a radiologist. After stent implantation, coil embolization was performed using 18 or 10 platinum 3D and helical coils (MicroVention-Terumo, Tustin, CA, USA) or Target Nano 360 and helical 10 coils (Stryker, Fremont, CA, USA). All centers in this study deployed all versions (A, B, C) of the LVIS Jr. stents, which showed a slight difference in radial force. Dual antiplatelet therapy continued for 3 months after discharge. Clinical outcome at 90 days was assessed using the modified Rankin Scale (mRS). All patients underwent follow-up imaging with time-of-flight or contrast-enhanced MR angiography (MRA) at 12 to 18 months post-procedure. The degree of aneurysm occlusion on immediate post-procedure angiogram and follow-up study was measured using the Raymond–Roy classification. The in-stent stenosis was defined as ≥50% luminal narrowing at follow-up imaging. There was no independent core lab to evaluate the imaging data.

Continuous variables and categorical variables are reported as mean (range) and percentage, respectively.

## 3. Results

### 3.1. Study Population and Aneurysm Characteristics

A total of 162 patients with 162 unruptured MCA aneurysms treated with the stent-assisted coiling were included in the study. There were 112 females (69.1%) and 50 males (30.9%) with a mean age of 57 years (range 29–79 years). The mean aneurysm size was 7.6 mm (range 2 to 37 mm) and mean aneurysm neck width was 5 mm (range 2 to 15 mm). In total, 125 aneurysms (77.2%) were <10 mm in size; 188 aneurysms (97.5%) were wide-necked. Wide-necked was defined as a neck width of ≥4 mm or a dome-to-neck ratio of ≤2. There were 154 bifurcation aneurysms (95.1%) and 8 sidewall aneurysms (4.9%) (Table 1).

### 3.2. Immediate Angiographic Outcome and Periprocedural Complications

**Figure 2:**
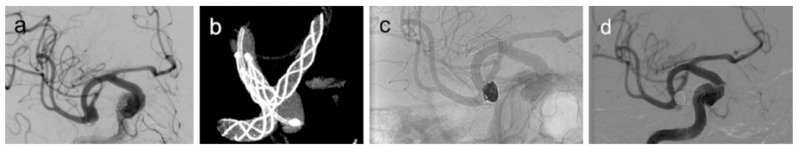
(**a**) A patient with a wide-necked right MCA bifurcation aneurysm; (**b**) the aneurysm was treated with the Y-stenting technique using two LVIS Jr. and jailed microcatheter technique visible on intraprocedural contrast-enhanced flat panel detector CT (MIP reconstruction); (**c**) final unsubtracted angiogram demonstrates the markers and radiopaque strands within the body of the stents; (**d**) post-procedural DSA showing complete occlusion of the aneurysm.

In 151 patients (93.2%), a single LVIS Jr. stent was implanted and in 11 patients (6.8%) a Y-stenting technique was used (Figure 2). The technical success rate of the procedure was 95.1%, as there were 8 cases of incomplete stent deployment. Two cases of incomplete stent deployment occurred in the subgroup of patients treated by Y-stenting. Aneurysm coiling was performed using a jailing technique in 100 patients (61.7%) and through stent struts in 62 patients (38.3%). The immediate postprocedural angiograms showed Raymond–Roy class 1 (complete occlusion) in 118 patients (72.8%), class 2 (neck remnant) in 23 patients (14.2%), and class 3 (residual sac) in 21 patients (13%). Periprocedural complications occurred in 14 patients (8.6%). There were 10 cases (6.2%) of in-stent thrombosis. Intravenous administration of glycoprotein IIb/IIIa antagonists resulted in complete thrombus resolution in all of these patients. Six patients (3.7%) experienced a transient ischemic attack (TIA). Five patients (3.1%) developed an ischemic stroke (two had hemiparesis and aphasia, one had aphasia and two had memory loss). Two strokes occurred in the subgroup of patients diagnosed with in-stent thrombosis. In 132 patients (81.5%), a vascular closure device was used to seal the femoral artery puncture. There were 2 cases (1.2%) of groin hematoma at puncture site. One of these patients developed a femoral artery pseudoaneurysm that was treated by ultrasound-guided compression with a good result. Femoral artery puncture site hemostasis in this patient was achieved by manual compression. There were no periprocedural complications in the subset of patients treated with the Y-stenting. The procedure-related mortality rate was 0%.

### 3.3. Follow-up Imaging and Clinical Outcomes

Follow-up imaging demonstrated complete occlusion in 10/23 aneurysms with neck remnant (43.5%) and in 11/21 aneurysms with residual sac (52.4%). Next, 4 of 21 aneurysms with residual sac (19%) improved to neck remnant; 3 of 118 initially occluded aneurysms (2.5%) and 4 or 23 aneurysms with neck remnant (17.4%) converted to residual sac. Neck remnant was observed in 4/118 initially occluded aneurysms (3.4%). The other 126 aneurysms (77.8%) remained stable. To summarize, follow-up imaging showed Raymond–Roy class 1 in 132 patients (81.5%), class 2 in 17 patients (10.5%), and class 3 in 13 patients (8%) (Table 2). Table 3 shows the aneurysm occlusion rate stratified by aneurysm size. In-stent stenosis was observed in 3 cases (1.9%). There were no other delayed complications. No delayed thromboembolic event occurred. All 162 patients had good clinical outcomes (mRS score 0–2) at 90 days post-procedure. Retreatment was performed in 7 aneurysms (4.3%).

## 4. Discussion

The development of stent-assisted coiling has allowed for a broader spectrum of intracranial aneurysms to be treated by the endovascular approach. The safety and efficacy of this endovascular technique has been demonstrated in numerous studies [8,9]. However, in a considerable number of papers, MCA aneurysms were underrepresented when compared to more proximal aneurysms. In a large series of 508 aneurysms by Chalouhi et al., they constituted only 2.9% of the analyzed cases. The same authors showed that this location was a statistically significant independent predictor of procedural complications and aneurysm recanalization [10]. Therefore, we believe that there is still a need to further investigate the results of stent-assisted coiling of intracranial aneurysms in this specific location.

To the best of our knowledge, we have reported the largest series of unruptured MCA aneurysms treated with the stent-assisted coiling. Several authors have reported on the safety and efficacy of the stent-assisted coiling of MCA aneurysms with the use of older generation neurovascular stents [11,12,13,14,15,16,17]. In a large study of 100 MCA aneurysms treated with the stent-assisted coiling by Johnson et al., the technical success rate of stent deployment was 100%. However, in 4% of the cases, the implanted device did not provide a sufficient coil mass support. Nine patients (9%) presented with new neurological symptoms following the procedure. The rates of major morbidity and mortality were 1% and 1%, respectively. Although initial angiographic results were not favorable, as only 48% of the aneurysms were totally occluded, follow-up imaging at 6 months showed total occlusion in 90.6%, residual neck in 3.5%, and residual sac in 5.9% of the aneurysms. It is worth noting that 85% of the treated aneurysms in this study were less than 10 mm in diameter and a considerable number of the lesions were not wide-necked, which could have contributed to the reported results [11]. Vendrell et al. achieved a successful stent deployment in 96.2% of the cases in the study of 52 unruptured MCA aneurysms with complex morphology. Interestingly, the authors observed a high grade of in-stent thrombosis (20%) that resulted in a minor stroke in 4 patients (8.5%), despite the prompt administration of abciximab. The high rate of thrombotic complication in this study may be attributed to the fact that the preprocedural antiplatelet therapy consisted only of a loading of dose of clopidogrel the day before the intervention. Midterm follow-up imaging showed total occlusion in 71%, residual neck in 12% and residual sac in 17% of the aneurysms. The recanalization rate was 14.6% and 10.4% of the aneurysms were qualified for retreatment. There were no procedure-related deaths and the rate of permanent neurologic morbidity was 4.3% [12]. Chen et al. also reported good angiographic results of 57 MCA aneurysms treated with the stent-assisted coiling. Follow-up imaging performed at 6–36 months post-procedure showed complete occlusion in 96% of the aneurysms [13]. In a more recent study by Hagen et al., which also included some new generation devices, the procedure-related complication rate of the stent-assisted coiling of unruptured MCA aneurysms was 7.4%. There was no significant difference in complication rates between MCA aneurysms treated with different endovascular techniques. The retreatment rate of the aneurysms treated with the stent-assisted coiling in this study was 5.9% [17].

All aneurysms in our registry were treated with the LVIS Jr. The LVIS Jr. is delivered through a 0.017-inch microcatheter and therefore may be deployed into small distal intracranial arteries. The key features of this device are its braided design with a high metal coverage and small pore size. These properties provide better coil mass support and improve flow diversion effect. Due to the compliant and flexible closed cell design of this device, a single stent may be used to treat the wide-necked bifurcation aneurysms with the so-called “shelf” technique. With this approach, a technically challenging Y-stenting can be avoided in many cases. In our series, the technical success rate of the Y-stenting was 81.8% (vs. 94% in patients treated with a single stent). One of the main safety concerns of the Y-stenting is a potentially higher risk of thromboembolic events due to increased metal density. However, we have not observed any periprocedural or delayed complications in this subset of patients. A recent meta-analysis by Cagnazzo et al. proved that the Y-stenting assisted coiling is an effective treatment for wide-necked intracranial aneurysms with a relatively low rate of treatment related complications [18]. Table 4 shows aneurysm occlusion rate stratified by number of implanted stents.

**Figure 3:**
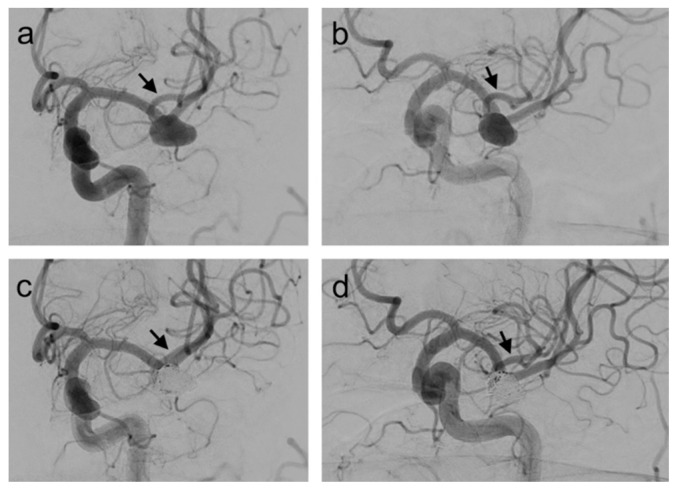
(**a**,**b**) A patient with a wide-necked left MCA bifurcation aneurysm. The aneurysm was treated with the stent-assisted coiling using the LVIS Jr. and coiling through the stent mesh; (**c**,**d**) post-procedural DSA showing complete occlusion of the aneurysm. Notice that the stent implantation resulted in the modification of the angle between the bifurcation branches (arrows).

Furthermore, in the cases of bifurcation aneurysms, the stent implantation results in the modification of the angle between the bifurcation branches (Figure 3). This vascular angular remodeling of the affected vessels changes local hemodynamics and thus may prevent aneurysm recanalization. Finally, the LVIS Jr. is resheathable up to approximately 80% of its deployment length, which facilitates repositioning. In their systematic review of the literature, Zhang et al. demonstrated the safety and efficacy of the LVIS and LVIS Jr. in the stent-assisted coiling of intracranial aneurysms [19]. However, many authors reported these combined results, including the outcomes of the procedures using the LVIS and those using the LVIS Jr. In addition to size, there are other significant differences in the construction of these devices. The LVIS Jr. has lower metal coverage and larger pore size than the LVIS (18% vs. 28% and 1.5 mm vs. 0.8 mm, respectively). We agree with Grossberg et al. that the results from these combined studies are not comparable to the studies investigating only the LVIS Jr. [20].

The current paper presents the largest series of stent-assisted coiling with the LVIS Jr. device. High technical success rate (95%) was observed in our study, which is similar to previous papers. The reported rate of occurrence of in-stent thrombosis is variable in the literature [20,21,22,23,24,25,26,27,28,29,30,31,32,33,34,35]. In another large series of aneurysms treated with the assistance of the LVIS Jr. by Shankar et al., it was observed in 14% of the cases. However, the overall mortality and permanent morbidity in this registry were low (1% and 3%, respectively) [28]. Wang et al. reported that LVIS/LVIS Jr. stents are associated with a higher incidence of in-stent thrombosis when compared to laser-cut stents [36]. In our series, the in-stent thrombosis was observed in 6.2% of the cases resulting in permanent neurologic deficits in 2 patients (1.2%). They presented with mRS scores of 1 and 2 at clinical follow-up at 90 days post-procedure. Platelet inhibition studies were not performed routinely in the participating centers, therefore the in-stent thrombosis could have been a manifestation of a resistance to clopidogrel in some patients. In general, we observed new neurological deficits following treatment in 11 patients (6.8%), six of which (3.7%) were transient and resolved within 24 h. There was no procedure-related death. Grossberg et al., in their study of 85 aneurysms, reported an in-stent thrombosis rate of 1.2%. The overall mortality and permanent morbidity in this series were 0% and 2.4%, respectively [20]. In a recent systematic review and meta-analysis by Cagnazzo et al., a subgroup analysis of aneurysms treated with low-profile braided stents has been performed. The technical success rate of the LVIS Jr. implantation was 96% and the procedure resulted in complete or near-complete aneurysm occlusion in 86% of the analyzed cases. The treatment-related complication rate was 7% with the ischemic and thromboembolic events being the most common. There was no case of treatment-related death in the investigated studies [35].

Despite the fact that our cohort consisted only of MCA aneurysms, which are considered technically challenging to treat by the endovascular approach, we achieved high immediate and long-term total occlusion rates, which are similar to, if not better than, those of other published LVIS Jr. series [20,21,22,23,24,25,26,27,28,29,30,31,32,33,34,35]. Follow-up imaging showed complete or near-complete aneurysm occlusion in 92% of the cases. Limitation of our study is the lack of independent core lab to evaluate the imaging data. Each participating investigator assessed the imaging data independently and sent the results to the lead investigator’s site in a standardized form. This might have led to the overestimation of the aneurysm total occlusion rate [37]. There is a significant discrepancy between our results and those of a recently published, multicenter, core lab adjudicated BRANCH study. The authors retrospectively evaluated the outcomes of endovascular treatment of unruptured MCA and basilar apex aneurysms. The core lab found that aneurysm total occlusion rate at follow-up was only 30.6%. However, there are some issues to consider when interpreting the results of this series. A total of 115 patients from 10 centers were enrolled during a 12-year period. This results in a considerably low number of patients per center per year [38]. Data from the literature shows that the outcomes of unruptured aneurysm coiling are better when a high-volume practitioner performs the procedure [39]. Shapiro et al. in their literature review also demonstrated the significance of operator experience in the overall success of stent-assisted coiling [8].

Our study has several limitations. We only provided the results from a short-term clinical follow-up at 90 days post-procedure. Furthermore, follow-up imaging was performed with MRA in all cases. Studies show that this imaging modality is highly accurate for the detection of aneurysm recanalization [40]. However, MRA has limitations in the diagnosis of in-stent stenosis. Signal loss from the metallic stent material hamper the evaluation for the patency of the parent artery. In our practice, whenever there is a suspicion of significant in-stent stenosis in follow-up MRA and patient is symptomatic, they are referred for DSA. We do not perform follow-up DSA routinely, due to its invasive nature, high costs, and radiation issues. As all three patients diagnosed with in-stent stenosis in our study were asymptomatic, they were not referred for DSA and were followed up with MRA. There were 12 patients (7.4%) with aneurysms ≤3 mm in our series. Patients treated with small aneurysms had previously ruptured another aneurysm, had risk factors that increased the risk of rupture, or had very high fear of living with an aneurysm without surgery.

## 5. Conclusions

The results of this multicenter registry show that the stent-assisted coiling of unruptured MCA aneurysms with the LVIS Jr. stent is safe and effective, with high immediate and long-term total occlusion rates. The mortality and morbidity rates of the procedure are acceptable.

## Figures and Tables

**Table 1:** Study population and aneurysm characteristics.

No of Patients	162
Age, mean (range)	57 (29–79)
Gender, *n* (%)	
Women	112 (69.1)
Men	50 (30.9)
Multiple aneurysms, *n* (%)	60 (37)
Family history of intracranial aneurysms, *n* (%)	7 (4)
Hypertension, *n* (%)	117 (72)
Smoking, *n* (%)	96 (59.3)
No of aneurysms treated	162
Aneurysm location, *n* (%)	
Bifurcation	154 (95.1)
Sidewall	8 (4.9)
Aneurysm size, mm, *n* (%)	
<7	86 (53.1)
7–10 mm	50 (30.9)
>10 mm	26 (16)
Neck width, mm, *n* (%)	
≥4	130 (80.2)
<4	32 (19.8)
Dome-to-neck ratio, *n* (%)	
≤2	145 (89.5)
>2	17 (10.5)

**Table 2:** Aneurysm occlusion rate immediately post-procedure and at 12–18 months follow-up imaging.

	Complete Occlusion (%)	Residual Neck (%)	Residual Sac (%)
Immediate postprocedural angiography	118 (72.8%)	23 (14.2%)	21 (13%)
12–18 months follow-up imaging	132 (81.5%)	17 (10.5%)	13 (8%)
Initial complete occlusion	111 (94.1%)	4 (3.4%)	3 (2.5%)
Initial residual neck	10 (43.5%)	9 (39.1%)	4 (17.4%)
Initial residual sac	11 (52.4%)	4 (19%)	6 (28.6%)

**Table 3:** Aneurysm occlusion rate stratified by aneurysm size.

Aneurysm Size	Immediate Postprocedural Angiography			12–18 Months Follow-up Imaging		
	Complete Occlusion (%)	Residual Neck (%)	Residual Sac (%)	Complete Occlusion (%)	Residual Neck (%)	Residual Sac (%)
<7 mm	69 (80.2%)	5 (5.8%)	12 (14%)	76 (88.4%)	6 (7%)	4 (4.6%)
7–10 mm	36 (72%)	6 (12%)	8 (16%)	43 (86%)	4 (8%)	3 (6%)
>10 mm	13 (50%)	12 (46.2%)	1 (3.8%)	13 (50%)	7 (27%)	6 (23%)

**Table 4:** Aneurysm occlusion rate stratified by number of implanted stents.

No. of Implanted Stents	Immediate Postprocedural Angiography			12–18 Months Follow-up Imaging		
	Complete Occlusion (%)	Residual Neck (%)	Residual Sac (%)	Complete Occlusion (%)	Residual Neck (%)	Residual Sac (%)
single stent	111 (73.5%)	20 (13.25%)	20 (13.25%)	122 (80.8%)	16 (10.6%)	13 (8.6%)
Y-stenting	7 (63.6%)	3 (27.3%)	1 (9.1%)	10 (90.9%)	1 (9.1%)	0 (0%)
